# 10 Years of GWAS in intraocular pressure

**DOI:** 10.3389/fgene.2023.1130106

**Published:** 2023-04-12

**Authors:** Xiaoyi Raymond Gao, Marion Chiariglione, Hélène Choquet, Alexander J. Arch

**Affiliations:** ^1^ Department of Ophthalmology and Visual Sciences, The Ohio State University, Columbus, OH, United States; ^2^ Department of Biomedical Informatics, The Ohio State University, Columbus, OH, United States; ^3^ Division of Human Genetics, The Ohio State University, Columbus, OH, United States; ^4^ Division of Research, Kaiser Permanente Northern California, Oakland, CA, United States

**Keywords:** intraocular pressure, GWAS, ExWAS, polygenic risk score, Mendelian randomization, underrepresented population

## Abstract

Intraocular pressure (IOP) is the only modifiable risk factor for glaucoma, the leading cause of irreversible blindness worldwide. In this review, we summarize the findings of genome-wide association studies (GWASs) of IOP published in the past 10 years and prior to December 2022. Over 190 genetic loci and candidate genes associated with IOP have been uncovered through GWASs, although most of these studies were conducted in subjects of European and Asian ancestries. We also discuss how these common variants have been used to derive polygenic risk scores for predicting IOP and glaucoma, and to infer causal relationship with other traits and conditions through Mendelian randomization. Additionally, we summarize the findings from a recent large-scale exome-wide association study (ExWAS) that identified rare variants associated with IOP in 40 novel genes, six of which are drug targets for clinical treatment or are being evaluated in clinical trials. Finally, we discuss the need for future genetic studies of IOP to include individuals from understudied populations, including Latinos and Africans, in order to fully characterize the genetic architecture of IOP.

## Introduction

The first genome-wide association study (GWAS) of intraocular pressure (IOP) was published in 2012 (van Koolwijk et al., 2012). Since then, numerous common genetic variants associated with IOP have been discovered (Hysi et al., 2014; Springelkamp et al., 2014; Choquet et al., 2017; Springelkamp et al., 2017; Gao et al., 2018; Khawaja et al., 2018; MacGregor et al., 2018). Recently, a study using whole-exome sequencing data on large-scale biobanks has also led to significant new gene discoveries in the genetic architecture of IOP, demonstrating the important contribution of rare variants to this glaucoma endophenotype (Gao et al., 2022). On the tenth anniversary of the first IOP GWAS, it is time to reflect on the progress that has been made in this field and to consider the future direction of the genetics of IOP.

IOP is the amount of fluid pressure in the eye which is mainly determined by the balance between aqueous humor production and drainage (Civan and Macknight, 2004; Machiele et al., 2022). Elevated IOP is a major risk factor for primary open-angle glaucoma (POAG), the most common form of glaucoma that affects around 90% of glaucoma patients (Lang, 2007). Currently, IOP is the only modifiable risk factor for glaucoma, and lowering IOP helps to prevent the onset and delay the progression of POAG (Collaborative Normal-Tension Glaucoma Study Group, 1998; Heijl et al., 2002; Kass et al., 2002). IOP can be influenced by many factors, such as time of the day (Qassim et al., 2020a), measurement techniques, such as the Goldmann applanation tonometer and the ocular response analyzer (Martinez-de-la-Casa et al., 2006), age and ethnic background (Klein et al., 1992; Wu and Leske, 1997; Baek et al., 2015), and genetics. Studies have found that the heritability of IOP ranges from 0.35 to 0.67, depending on the study design (Klein et al., 2004; Chang et al., 2005; van Koolwijk et al., 2007; Carbonaro et al., 2009; Zheng et al., 2009; Sanfilippo et al., 2010; Asefa et al., 2019). Identifying genetic factors that contribute to IOP aid in uncovering the biological mechanisms regulating this trait (Ojha et al., 2013; Xu et al., 2021), which provides new management avenues for IOP and POAG.

GWASs have identified over 190 genetic loci associated with IOP (Choquet et al., 2017; Gao et al., 2018; Khawaja et al., 2018; MacGregor et al., 2018), demonstrating the contribution of common genetic variants to this trait. Additionally, these studies have shown that there is a strong bivariate genetic correlation between IOP and POAG ranging from 0.49 to 0.71 (Aschard et al., 2017; MacGregor et al., 2018) and a significant polygenic overlap between these two traits (Hysi et al., 2014; Khawaja et al., 2018). While numerous loci have been associated with IOP, these common variants typically have small effect sizes, contrary to those seen in rare variants, which can have large effect sizes (Gao et al., 2018; Gao et al., 2022). The role of rare genetic variants in IOP was recently reported in a large-scale exome-wide association study (ExWAS) that identified 40 novel genes associated with IOP (Gao et al., 2022).

Over the last 10 years, GWASs and ExWAS have provided strong evidence that IOP is a polygenic trait and a powerful endophenotype for POAG. These studies have also revealed valuable biological insights, pleiotropic effects, and potential drug targets associated with IOP genetic loci. The results of these studies have been applied to the development of polygenic risk scores (PRSs), which could potentially be used to stratify and screen for POAG risk in a population using IOP information (Khawaja et al., 2018; MacGregor et al., 2018; Gao et al., 2019; Qassim et al., 2020b). Moreover, the results of these studies have been applied to the development of genetic instruments that may be used in Mendelian randomization (MR) studies to better understand the nature of the relationships between eye traits and conditions (Han et al., 2020; Hysi et al., 2020; Choquet et al., 2022). In this review, we summarize these findings and discuss the potential future of GWASs in the study of IOP.

## GWAS and ExWAS

GWASs have revolutionized the field of complex diseases and traits genetics over the past 17 years (Visscher et al., 2017; Tam et al., 2019). They involve examining the association between diseases or traits and hundreds of thousands (Klein et al., 2005) to millions of densely spaced single nucleotide polymorphisms (SNPs) (Gao and Edwards, 2011). These studies do not require any prior biological knowledge and are therefore an agnostic method for identifying the genetic effects of complex human diseases and traits. They are based on the assumption that densely genotyped common variants (minor allele frequency [MAF] ≥ 1%) will have sufficient statistical power to detect associations. This approach has been successful in numerous cases for mapping small genomic regions to diseases and traits (Welter et al., 2014). Many of these regions would not have been considered good candidates for targeted genotyping based on biological knowledge or previous evidence of linkage. Most of the findings from GWASs are collected in the GWAS Catalog, a database of all published GWASs maintained by the National Human Genome Research Institute (NHGRI) and the European Bioinformatics Institute (EBMI-EBI) (MacArthur et al., 2017). A standardized significance threshold of a *p*-value less than 5 × 10^−8^ has been adopted by the genetics community as the genome-wide level of significance, which is based on the assumption of one million independent pieces of genetic information in the human genome (Risch and Merikangas, 1996; Pe’er et al., 2008).

Each individual GWAS can have a limited sample size, which affects its statistical power. Additionally, genetic association signals that are identified need to be independently replicated in order to be considered reliable. To overcome these limitations, researchers often use genome-wide association meta-analysis (Willer et al., 2010; McGuire et al., 2021), which combines the results of multiple GWAS studies, and genotype imputation (Li and Abecasis, 2006; Marchini et al., 2007), which can infer ungenotyped variants from known data. Together, these methods can provide a more comprehensive and robust analysis of the genetic basis of a particular trait or disease.

More recently, other genome-wide studies, including whole-exome and whole-genome sequencing studies have been conducted in parallel of GWASs to assess rare variants associations and their roles in the genetic causes of diseases/traits. ExWAS employs whole-exome sequencing data that focuses on specific parts of the genome that encodes proteins, called exons, and allows for changes within such regions to be identified and analyzed. To address the relatively low statistical power issues in rare-variant analysis, researchers have designed many collapsing or gene-based methods (Li and Leal, 2008; Madsen and Browning, 2009; Wu et al., 2010; Wu et al., 2011; Lee et al., 2012a; Lee et al., 2012b; Zhou et al., 2018; Zhou et al., 2020).

## Study inclusion criteria

To identify previously published IOP GWAS and ExWAS papers, we queried two websites: GWAS Catalog and PubMed, using the keywords “intraocular pressure” and “exome intraocular pressure,” respectively. We found 28 studies in the GWAS Catalog and three in PubMed. We then manually curated the search results to focus on studies with IOP as the main phenotype. Finally, we excluded studies that did not report novel significant IOP loci. As a result, Table 1 includes the 14 studies (GWAS and ExWAS) that were selected based on these two criteria: 1) IOP as the target phenotype of GWAS analyses; and 2) reported novel findings being genome-wide or exome-wide significant, either single variant (*p* < 5.0 × 10^−8^) or gene-based (*p* < 2.5 × 10^−6^), over the past 10 years. One study (Ozel et al., 2014) that reported borderline genome-wide significance (*p* = 8 × 10^−8^) was included in Table 1 as well. These criteria excluded two IOP studies (Chen et al., 2015; Chakraborty et al., 2021) that reported suggestively significant findings (*P* ∼ 5.0 × 10^−5^ or *P* ∼ 5.0 × 10^−6^).

**TABLE 1:** Genome-wide and exome-wide association studies of intraocular pressure in the last 10 years.

Study name	Year	Novel loci	Sample size	Population	Total association count	Replication sample size	Number of single-variants tested
GWAS							
van Koolwijk et al.	2012	2	11,972	European	2	7,482	2.5 Million
Blue Mountains Eye Study (BMES);Wellcome Trust Case Control Consortium 2 (WTCCC2)	2013	1	2,175	European	1	4,866	6.2 Million
Ozel et al.	2014	1	6,000	European	1	-	2.54 Million
Nag et al.	2014	1	2,774	European	1	22,789	1.87 Million
Hysi et al.	2014	4	35,296	European (*n* = 27,558)	8	99,844	-
				Asian (*n* = 7,738)			
Springelkamp et al.	2015	1	8,105	European	1	7,471	1000 Genomes phase 1 imputation^a^
Springelkamp et al.	2017	1	37,930	European (*n* = 29,578)	10	47,833	8 Million
				Asian (*n* = 8,352)			
Choquet et al.	2017	40	69,756	European (*n* = 56,819)	47	Springelkamp et al. (2017) summary statistics, 37,930	15 Million
				Latino (*n* = 5,748)			
				Asian (*n* = 5,119)			
				African (*n* = 2,070)			
Gao et al.	2018	145^b^/103^c^	115,486	European	191	Springelkamp et al. (2017) summary statistics, 37,930	11.9 Million
Khawaja et al.	2018	68	139,555	European	112	6,595 (EPIC-Norfolk)	9.1 Million
						29,578 (IGGC)	
MacGregor et al.	2018	85	103,914 (UKB) and 29,578 (IGGC)	European	106	-	40 Million
Huang et al.	2019	17	8,552	Chinese	21	2,981	1000 Genomes phase 1 imputation^a^
Simcoe et al.	2020	3	102,407	European	3	6,599 (EPIC-Norfolk)	590,896
						331,682 (UKB)	
ExWAS							
Gao et al.	2022	40	110,260	European (*n* = 98,674)	46	FinnGen summary statistics, 340,048	15 Million
				African (*n* = 3,286)			
				Asian (*n* = 3,755)			
				Others (*n* = 4,545)			

^a^ Variants were imputed from the 1000 Genomes Project phase 1 reference panel, but the exact number of variants was not reported.

^b^ Genetic loci identified using genotyped and imputed variants.

^c^ Genetic loci identified using directly genotyped variants.

UKB: UK Biobank; IGGC: the International Glaucoma Genetics Consortium.

## GWAS of IOP

van Koolwijk et al. (2012) reported the first GWAS on IOP in 2012. They used 11,972 participants from four cohorts in The Netherlands, conducted linear regression analysis in each cohort, and then performed meta-analyses. They further carried out replication using cohorts from UK, Australia, Canada, and The Wellcome Trust Case Control Consortium 2 (WTCCC2)/Blue Mountains Eye Study (BMES). Variants rs11656696 at *GAS7* and rs7555523 at *TMCO1* were significantly associated at the genome-wide level with IOP and were also associated with POAG. After 2012, multiple groups continued to rely on meta-analysis of GWASs as their primary method for identifying genetic loci related to IOP.

Researchers from The Blue Mountains Eye Study and The Wellcome Trust Case Control Consortium 2 (2013) identified rs59072263, a common variant between *GLCCI1* and *ICA1* at 7q21, which had a combined *p* = 1.10 × 10^−8^ in a meta-analysis of three cohorts: BMES (*n* = 2,175), EPIC-Norfold (n = 2,461), and TwinsUK (*n* = 2033). Ozel et al. (2014) performed a GWAS and a meta-analysis of IOP in participants of European ancestry from three cohorts, i.e., the NEI Glaucoma Human Genetics Collaboration (NEIGHBOR), GLAUcoma Genes and ENvironment (GLAUGEN) study, and a subset of the Age-related Macular Degeneration-Michigan, Mayo, Age-Related Eye Disease Studies (AREDS) and Pennsylvania study, totaling >6,000 individuals. Although no association with IOP reached genome-wide significance in any single cohort, the combination of results from all cohorts in a meta-analysis revealed a borderline genome-wide significant association at the *TMCO1* locus (rs7518099-G, *p* = 8.0 × 10^−8^). Nag et al. (2014) reported that rs2286885 within *FAM125B* was associated with IOP in the TwinsUK cohort (N = 2,774) and replicated the signal in 12 independent replication cohorts of European ancestry (combined *n* = 22,789). Hysi et al. (2014) carried out a large-scale meta-analysis of 18 cohorts from the International Glaucoma Genetics Consortium (IGGC, *n* = 35,296, 27,558 individuals of European ancestry and 7,738 individuals of Asian ancestry) and found four new IOP loci, rs6445055 in *FNDC3B*, rs2472493 near *ABCA1*, rs8176693 in *ABO*, and rs747782 on 11p11.2, among which loci, i.e., *ABCA1* (rs2472493), *FNDC3B* (rs6445055), and 11p11.2 (rs12419342), were also associated with POAG risk in four independent cohorts (all of European ancestry, 4,284 cases and 95,560 controls). In the meta-analysis of the Rotterdam Study I and II cohorts (*n* = 8,105 participants), Springelkamp et al. (2015) identified three SNPs in *ARHGEF12* that reached genome-wide significance. In a following larger meta-analysis of individuals of European and Asian descent (*n* = 37,930), Springelkamp et al. (2017) identified rs55796939 near *ADAMTS8* as a new locus for IOP. Using multiple longitudinal IOP measurements from electronic health records, Choquet et al. (2017) conducted a large multi-ethnic meta-analysis of IOP in the Kaiser Permanente GERA cohort, combining 69,756 individuals of European (*n* = 56,819), Hispanic/Latino (*n* = 5,748), East Asian (*n* = 5,119), and African (*n* = 2,070) ancestry, and reported 40 novel loci. Around 2017, large and multiethnic biobank datasets gradually became accessible to general researchers and, significantly boosted genetic discoveries related to IOP.

In 2018, with the advent of the biobank era, three groups reported genetic loci for IOP using the large UK Biobank prospective cohort dataset (Allen et al., 2014; Sudlow et al., 2015). Gao et al. (2018) described a GWAS of IOP using 115,486 European UKB participants and identified 103 and 145 novel loci using directly genotyped SNPs and an imputed genetic dataset, respectively. In addition to uncovering common variants, Gao et al. (2018) also reported low-frequency variant (MAF in the range of 0.005–0.01) associations with IOP, including rs28991009 in *ANGPTL7*. rs28991009 was subsequently studied in another report by Tanigawa et al. (2020). Khawaja et al. (2018) performed a meta-analysis of 139,555 European participants from UKB (*n* = 103,382), the International Glaucoma Genetics Consortium (IGGC) (*n* = 29,578) and EPIC-Norforlk (*n* = 6,595), and identified 68 novel genomic loci associated with IOP. MacGregor et al. (2018) identified 85 novel loci for IOP using a combined analysis of 133,492 participants from UKB (*n* = 103,914) and results from IGCC (*n* = 29,578). The large number of novel IOP loci identified by independent groups clearly demonstrated the power of the large-scale UKB dataset.

In the 2 years following 2018, two studies reported common-variant associations for IOP. Huang et al. (2019) reported 17 newly identified loci for IOP from a GWAS of 8,552 Chinese participants. In contrast to previous studies that explored autosomal SNPs, Simcoe et al. (2020) performed association analyses across the X chromosome using 102,407 participants from UKB and identified three loci, located within or near *MXRA5* (rs2107482), *GPM6B* (rs66819623), and *NDP*/*EFHC2* (rs12558081), associated with IOP.

**FIGURE 1:**
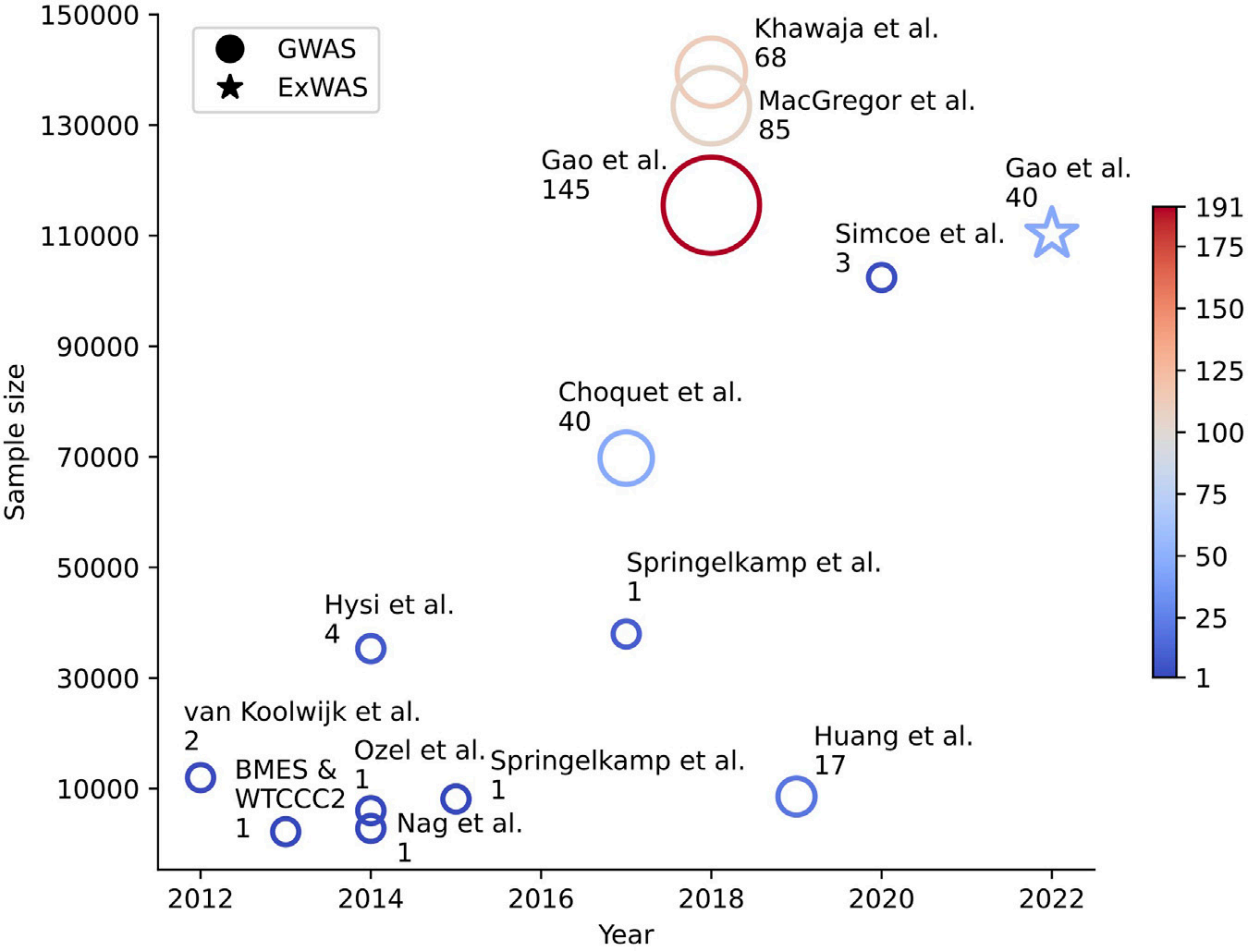
Number of novel intraocular pressure loci and study sample size in the last 10 years. Genetic association studies for IOP published in the past 10 years are represented by either (a) a circle for GWAS or (b) a star for ExWAS. The x-axis shows the year the study was published, and the y-axis shows the sample size of the study. The color and size of each plotted icon are proportional to the total number of loci and the number of novel loci discoveries that were reported in the corresponding study, respectively.

Figure 1 shows the relationship between the number of novel IOP loci identified and the sample sizes used in 13 previously published GWASs and one ExWAS (described in more details in the next section) in the last 10 years. With larger sample sizes, typically more novel loci are identified. For instance, for a cohort size of about 12,000–35,000 European individuals, only one to four novel IOP loci were identified from GWASs. When the sample size increased to around 110,000 European individuals, more than 100 novel loci were identified, which represent a significant increase in the number of identified loci compared to studies with less than 40,000 individuals. However, for not well studied populations, such as East Asian, even less than 9,000 individuals generated 17 novel loci. This may indicate some genetic differences of IOP among different ethnic groups. For the three reports in 2018, i.e., Gao et al. (2018), Khawaja et al. (2018), MacGregor et al. (2018), different numbers of novel IOP loci were identified among different research groups, though there was a large overlap of the UKB sample used, possibly due to different analytic approaches, including MAF cutoff, phenotype definition, and number of principal components of genetic ancestry adjusted for.

## ExWAS of IOP

ExWAS is similar to, but different from, a common-variant GWAS. It focuses on coding regions and a different set of genetic variants, specifically rare ones typically with MAF less than 0.01, which represent a new avenue for IOP genetics research. In a very recent study, Gao et al. (2022) reported the largest rare-variant study of IOP to date using whole-exome sequences of 110,260 UKB participants. In addition to confirming known IOP genes, Gao’s group identified 40 novel genes harboring rare variants associated with IOP, including *BOD1L1*, *ACAD10*, *HLA-B*, *ADRB1*, *PTPRB*, *RPL26*, *RPL10A*, *EGLN2*, and *MTOR*. This study demonstrated the power of including and aggregating rare variants in gene discovery. About half of the identified IOP genes were also found to be associated with glaucoma phenotypes in UKB and the FinnGen cohort, a large biobank study focused on the population of Finland (Kurki et al., 2022). Most of the novel rare variants associated with IOP in Gao et al. (2022)’s study showed large effect sizes, which is consistent with the pattern that rare variants can show much larger effect sizes than common variants observed in many other studies (Gorlov et al., 2011; Zuk et al., 2014; Forgetta et al., 2020; Van Hout et al., 2020).

## Biological insights

The above GWAS and ExWAS studies of IOP provided invaluable biological insights into both IOP and POAG. For example, van Koolwijk et al. (2012) found that *GAS7* and *TMCO1* are highly expressed in glaucoma-related ocular tissues, such as ciliary body, trabecular meshwork, lamina cribrosa, optic nerve, and retina. Hysi et al. (2014) found that *FNDC3B* and *ABCA1* also showed association with POAG and both genes were expressed in most ocular tissues. In Choquet et al. (2017), Choquet et al. (2018), functional studies support IOP-related influences of *FMNL2* and *LMX1B*, with certain *LMX1B* mutations causing high IOP and glaucoma resembling POAG in mice. In Gao et al. (2018), the top five Reactome pathways associated with IOP included the olfactory signaling pathway, defective *B3GALTL* causing Peters-plus syndrome, O-glycosylation of TSR domain-containing proteins, *ABC* transports in lipid homeostasis, and extracellular matrix organization. The loci reported by Khawaja et al. (2018) suggest that angiopoietin-receptor tyrosine kinase signaling, lipid metabolism, mitochondrial function, and developmental processes play a significant role in the risk of elevated IOP. Additionally, 14 of these associations were significantly associated with POAG after correction for multiple comparisons. MacGregor et al. (2018) studied the expression of genes at the newly identified IOP loci that were also associated with glaucoma in various human ocular tissues, including the corneal epithelium, corneal stroma, corneal endothelium, trabecular meshwork, ciliary body pigmented epithelium, neurosensory retina, optic nerve head, and optic nerve. They found that the expression of their newly associated genes was more enriched in the trabecular meshwork than other ocular tissues. MacGregor et al. further used FANTOM5 Cap Analysis of Gene Expression data and found evidence of correlation between enhancers with associated SNPs and the promoters of nine genes, including *PTPN1*, *BCLAF1*, and *GAS7*, in stromal and eye tissues. Nair et al. (2021) showed that mice deficient in GLIS1 developed chronically elevated IOP and GLIS1 impacts the expression of several other IOP and glaucoma-related genes, including *MYOC* and *CYP1B1*.

Numerous IOP genes also showed apparent pleiotropic nature (Choquet et al., 2017; Gao et al., 2018). Pleiotropy is the phenomenon in which a single gene or genetic variant has multiple effects on different traits (Stearns, 2010; Solovieff et al., 2013). Pleiotropy is important because it helps explain how a single genetic change can have multiple effects on an organism. It also helps to explain why certain traits and conditions may be inherited together, even if they seem unrelated. In addition to glaucoma risk, Choquet et al. (2017) reported that several their own IOP loci are also associated with cup area, central corneal thickness, and Axenfeld-Rieger syndrome. Gao et al. (2018) studied the pleiotropic effects of 671 variants (from directly genotyped variants found in 149 unique loci) using the GWAS catalog. Many neurological disorders associated with eye diseases were directly linked to the included SNPs, such as primary open-angle, primary angle closure, and high-pressure glaucoma, as well as age-related macular degeneration. In addition, ocular parameters such as central corneal thickness, axial length, optic cup area, and iris characteristics were mapped. The SNPs were also matched to digestive and immune disorders, cancer, and cardiovascular and hematological measurements, including blood pressure, body mass index, and type 2 diabetes. Pleiotropy undoubtedly plays an important role in furthering our understanding of human biology and disease (Gao and Huang, 2019), including IOP and glaucoma. Studying the pleiotropic nature of human traits can provide new insights into disease prevention and treatment (Gao, 2020), e.g., drugs that have been approved for the treatment of one disease could be repurposed for the management of IOP based on information about pleiotropy. This may lead to the discovery of novel uses for existing drugs.

## Drug targets

One of the goals of GWAS/ExWAS is to facilitate drug target discoveries, which has been the endeavor of many pharmaceutical companies. Drug candidates that have genetics support are twice as likely to be successful as those without genetics support (Nelson et al., 2015). Focused analyses of *CAV1*/*CAV2* revealed their association with IOP and replicated the previously reported associations with POAG in both effect size and direction (Ozel et al., 2014). Knockout mice exhibit elevated IOP and decreased outflow facility, demonstrating the direct role for *CAV1* in IOP homeostasis (Elliott et al., 2014). The extracellular matrix (ECM) was also shown to be associated with IOP (Choquet et al., 2017; Gao et al., 2018; MacGregor et al., 2018). ECM plays an important role in regulating the outflow of aqueous humor and may be a promising target for new therapies, such as those targeting the rho kinase, nitric oxide, adenosine A_1_, prostaglandin EP_4_, and potassium channel pathways involved in the conventional outflow of aqueous humor (Prasanna et al., 2016). Manipulating the ECM in the aqueous outflow pathway impacts IOP in genetic knockouts (Vranka et al., 2015). Furthermore, *ANGPTL7* was shown to modulate the trabecular meshwork’s ECM and the response of this tissue to steroids (Comes et al., 2011) and may serve as a good candidate for glaucoma therapy (Borrás, 2017). Six genes, namely, *ADRB1*, *PTPRB*, *RPL26*, *RPL10A*, *EGLN2*, and *MTOR*, out of Gao et al. (2022)’s gene-based investigation have existing therapeutic molecular targets. The most notable one, *ADRB1*, is the target of cardiovascular and glaucoma drugs, including the broad class of glaucoma drugs targeting the beta-adrenergic receptor antagonists, or beta-blockers, known to lower IOP. The other five genes are targets in many clinical trials involving razuprotafib (targeting *PTPRB*), ataluren, ELX-02, MT-3724 (targeting *RPL26* and *RPL10A*), roxadustat, daprodustat, vadadustat (targeting *EGLN2*), and perhexiline (targeting *MTOR*), providing candidates for drug repurposing for possible glaucoma treatment.

## Applications of GWAS/ExWAS results

In addition to the biological insights that we can gain from all these GWAS and ExWAS studies, there are two other major categories of applications, i.e., PRS and MR, utilizing the summary statistics from GWAS/ExWAS studies to make powerful predictions, e.g., to stratify individuals into high and low risk groups, and to infer possible causal effects.

### Polygenic risk scores

Similarly to the three IOP GWASs reported in 2018, studies by Khawaja et al. (2018), MacGregor et al. (2018), and Gao and Fan (2018) also explored the use of IOP PRS or SNPs to predict glaucoma. Both Khawaja et al. (2018), and MacGregor et al. (2018) used *p* < 5 × 10^−8^ to select IOP SNPs for predicting glaucoma. Khawaja et al. used a regression-based model instead of PRS and got an area under the receiver operating characteristic curve (AUC) of 0.74. MacGregor et al. used an allele-score approach by combining the IOP and vertical cup disc ratio (VCDR) allele scores for their glaucoma prediction. Individuals in the top 5%, 10%, and 20% of their allele scores were at significantly increased risk of POAG compared to those in the bottom 5%, 10%, and 20% (OR = 7.8, 5.6, and 4.2, respectively). Gao and Fan (2018) tested a grid of *p*-value cutoffs for selecting SNPs, such as 0.01, 0.001, 10^−4^, 5 × 10^−5^, and 5 × 10^−8^. They found that 5 × 10^−5^ gave better prediction accuracy for IOP and glaucoma than the 5 × 10^−8^ cutoff; Gao et al. (2019) observed significant associations between the IOP PRS (weighted) and IOP, with increasing PRS associated with higher IOP. Moreover, the PRS explained an additional 4% of the variation in IOP. They also identified significant associations between the IOP PRS and glaucoma, with study participants in the upper PRS quintiles experiencing greater odds of glaucoma (OR = 6.34) compared to those in the lowest quintile. Overall, the weighted PRS yielded a significant increase (*p* = 6.2 × 10^−222^) in the AUC to 0.77 compared to the model using age, sex, body mass index, systolic blood pressure, and type 2 diabetes. Furthermore, they observed similar results for the unweighted PRSs. IOP PRSs were also found to be associated with IOP readings outside clinic office hours, maximum IOP, glaucoma severity, and glaucoma treatment intensity (Qassim et al., 2020a; Qassim et al., 2020b). Gao et al. (2022) further constructed a rare-variant IOP PRS in their whole-exome sequencing study and showed that it is significantly associated with glaucoma in independent individuals.

### Mendelian randomization

GWAS results and PRSs are also used in other types of studies, such as MR studies, which observe the causal effects of an exposure to a specific external factor or a disease outcome based on variation in the population genome. They play an important role in our understanding of how external factors influence the development of a disease in an individual based on their specific genetic makeup. Kim et al. (2021) carried out MR analyses using the UKB dataset and assessed whether genetic loci linked to coffee consumption were associated with IOP. By using a PRS combining 111 IOP genetic variants, they were able to observe the interactions between the cohort’s genomes and diets for over 121,000 individuals. They observed that coffee, tea, and caffeine consumption were weakly associated with lower IOP, and that these exposures had no association with glaucoma. However, the association between caffeine intake and IOP was modified by an IOP PRS, such that higher caffeine intake was positively associated with both IOP and glaucoma prevalence, but only among individuals with the highest genetic susceptibility to elevated IOP. Hysi et al. (2020) used variants of IOP as instruments and explored the relationship between refractive error and IOP. They found that IOP predicts a decrease in the spherical equivalent of diopters (more myopic). Choquet et al. (2022) conducted a two-sample MR study to evaluate the nature of the relationship between myopic refractive error and POAG and performed a multivariable MR analysis to adjust for the potential effect of IOP. Han et al. (2020) used a two-sample MR and found evidence of a potential causal inference for the associations of myopia and IOP with retinal detachment.

## Genetic association studies of IOP in underrepresented populations

Genetic association studies of IOP in underrepresented populations are rather scarce. Here, underrepresented populations are defined as subgroups that have low representation relative to their numbers in the general population based on the definition described by the National Center for Advancing Translational Sciences of NIH (https://toolkit.ncats.nih.gov/glossary/underrepresented-population/). As such, the following groups are considered underrepresented: African Americans, Hispanics or Latinos, American Indians Alaskan Natives, and Native Hawaiians and other Pacific Islanders. Instead of carrying out ancestry-specific studies, most previous association studies embedded underrepresented populations in studies with a large number of European individuals (Choquet et al., 2017; Gao et al., 2022), possibly due to the smaller sample size of each individual underrepresented cohort. Choquet et al. (2017) used a meta-analysis approach to include underrepresented population samples to increase the overall sample size in their common variant association with IOP analysis. Gao et al. (2022) used a pan-ancestry approach by pooling all samples together and applying mixed-effect models that accounted for both principal components of genetic ancestry and genetic subpopulations in their rare variant analysis. After IOP genetic loci were identified in the overall combined multiethnic meta-analysis or sample, the loci were then explored in individual ethnicity groups (Choquet et al., 2017; Gao et al., 2022). To the best of our knowledge, only one study examined the association between IOP and genetic ancestry in a standalone Latino cohort (Nannini et al., 2016). Using linear regression analyses, Nannini et al. (2016) found that African ancestry was significantly associated with higher IOP in Latinos. After accounting for age, sex, body mass index, systolic blood pressure, central corneal thickness, and type 2 diabetes, this association remained significant. They also found that the association between African ancestry and IOP was modified by a significant interaction with hypertension, such that hypertensive individuals experienced a greater increase in IOP with increasing African ancestry. This study demonstrated for the first time that African ancestry and its interaction with hypertension are associated with higher IOP in Latinos.

## Discussion

Since the first success of GWAS in human genetics (Klein et al., 2005), GWASs have become a widely used tool in genetic epidemiology (Gao and Edwards, 2011). These studies have led to the identification of many genetic variants that are associated with a variety of human diseases and traits, including IOP and glaucoma. The use of GWASs has greatly enhanced our ability to search for genetic contributions to complex traits. Due to the need for high statistical power, researchers often employ meta-analysis to combine the results of multiple GWAS studies. The availability of biobank datasets, especially UKB with half a million participants, also propelled genetic studies to another level of discoveries in both common and rare variants. Through ExWASs, large effect-size rare variants for IOP have begun to be unveiled. Such sequencing approaches will become more prevalent in the research world in the years to come. Not only have we seen a much deeper level of biological insights, including pleiotropy, but also have researchers used IOP PRS to help predict glaucoma and genetic instruments for IOP in MR studies for inferring causal relationships with other eye conditions.

Despite all the discoveries that have been made, the majority of the GWASs of IOP were done in individuals of European (95.6%) and Asian (3.1%) descents. Some efforts have been made to include individuals of non-European descent, such as the studies conducted by Choquet et al. (2017); Gao et al. (2022). However, the underrepresented population specific information is still largely unknown. Genetic discoveries in standalone underrepresented population are still in scarcity, with only one Latino-specific genetic association study published so far. Presently, PRSs work mostly in European individuals since most GWASs were done in European samples and do not transfer well to other underrepresented populations. PRS widespread use can create health disparities if this continues (Martin et al., 2019). It would be interesting to compare the discovery of genetic variants associated with a particular trait or disease in a sample of European individuals to that of a sample of individuals of African descent or Latino individuals, with the same sample size, for example, of 100,000 individuals. This comparison could provide insight into potential differences and similarities in the genetic basis of the trait or disease between these populations, which can have implications for the development and application of genetic testing and personalized medicine. Equity, diversity, and inclusion are critically important in all aspects of our life, including genetic research. Efforts to diversity are being made to address the significant imbalance in this field (Denny and Collins, 2021). The All of Us research program, a part of the National Institutes of Health, is working to build an inclusive, diverse database by inviting individuals from all backgrounds to participate (The All of Us Research Program Investigators, 2019). They have over 550,000 enrolled participants, with over 387,000 having completed the first steps to integrate the research program. Over 50% of their participants are from an ethnic minority, and over 80% correspond to a group underrepresented in research.

At the start of a new decade of IOP GWASs, what can we expect to see next? It is possible that GWASs by genotyping arrays will be replaced by GWASs by sequencing as the cost of sequencing continue to decrease. The use of whole-genome sequencing in GWASs is almost certain to yield unexpected discoveries, similar to what agnostic GWASs have already shown. Additionally, advancements in artificial intelligence may transform how we analyze and understand human genetics (Gao et al., 2020). These genetic discoveries are likely to be applied to individual patients of all backgrounds to aid in prevention, diagnosis and treatment.
